# Clinicopathological analysis of *NEK1* variants in amyotrophic lateral sclerosis

**DOI:** 10.1111/bpa.13287

**Published:** 2024-07-10

**Authors:** Olivia M. Rifai, Fergal M. Waldron, Danah Sleibi, Judi O'Shaughnessy, Danielle J. Leighton, Jenna M. Gregory

**Affiliations:** ^1^ Centre for Discovery Brain Sciences University of Edinburgh Edinburgh UK; ^2^ Department of Neurology Center for Motor Neuron Biology and Disease, Columbia University New York New York USA; ^3^ Institute of Medical Sciences University of Aberdeen Aberdeen UK; ^4^ Department of Chemistry University of Edinburgh Edinburgh UK; ^5^ Department of Neurology University of Glasgow Glasgow UK; ^6^ School of Psychology & Neuroscience University of Glasgow Glasgow UK; ^7^ Euan MacDonald Centre for Motor Neuron Disease Research University of Edinburgh Edinburgh UK

**Keywords:** ALS, genetics, NEK1, neuropathology, post‐mortem

## Abstract

Many genes have been linked to amyotrophic lateral sclerosis (ALS), including never in mitosis A (NIMA)‐related kinase 1 (NEK1), a serine/threonine kinase that plays a key role in several cellular functions, such as DNA damage response and cell cycle regulation. Whole‐exome sequencing studies have shown that *NEK1* mutations are associated with an increased risk for ALS, where a significant enrichment of *NEK1* loss‐of‐function (LOF) variants were found in individuals with ALS compared to controls. In particular, the p.Arg261His missense variant was associated with significantly increased disease susceptibility. This case series aims to understand the neuropathological phenotypes resulting from *NEK1* mutations in ALS. We examined a cohort of three Scottish patients with a mutation in the *NEK1* gene and evaluated the distribution and cellular expression of *NEK1*, as well as the abundance of phosphorylated TDP‐43 (pTDP‐43) aggregates, in the motor cortex compared to age‐ and sex‐matched control tissue. We show pathological, cytoplasmic TDP‐43 aggregates in all three *NEK1*‐ALS cases. NEK1 protein staining revealed no immunoreactivity in two of the *NEK1*‐ALS cases, indicating a LOF and corresponding to a reduction in *NEK1* mRNA as detected by in situ hybridisation. However, the p.Arg261His missense mutation resulted in an increase in *NEK1* mRNA molecules and abundant NEK1‐positive cytoplasmic aggregates, with the same morphologic appearance, and within the same cells as co‐occurring TDP‐43 aggregates. Here we show the first neuropathological assessment of a series of ALS cases carrying mutations in the *NEK1* gene. Specifically, we show that TDP‐43 pathology is present in these cases and that potential NEK1 LOF can either be mediated through loss of *NEK1* translation or through aggregation of NEK1 protein as in the case with p.Arg261His mutation, a potential novel pathological feature of NEK1‐ALS.

## Graphical abstract

Illustration of the structural and functional consequences of ALS‐associated NEK1 mutations in our cohort.

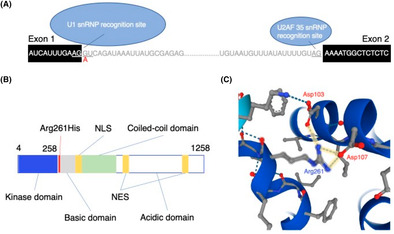

## 1 INTRODUCTION

Amyotrophic lateral sclerosis (ALS) is a neurodegenerative disease characterised by progressive loss of motor function, with death occurring within three to 5 years of onset [1]. There exists a genetic, clinical, and pathological overlap between ALS and frontotemporal dementia (FTD) [2], whereby several genes (e.g., *C9orf72, TARDBP, TBK1*) [3] have been associated with both conditions. Recently, mutations in *NEK1* have been shown to confer susceptibility to ALS in whole exome sequencing studies [4, 5, 6], though the clinical and pathological phenotypes associated with *NEK1* variants are currently poorly understood. To date, there are very few studies that have pathologically examined *post‐mortem* tissue from these rare cases. One study has examined NEK1 using patient tissue [7] but no study has examined TDP‐43 and NEK1 pathology in the same cases.

NEK1, or (never in mitosis gene‐A)‐related kinase 1, is a serine/threonine kinase involved in the formation of the microtubule‐based primary cilium [8]. NEK1 is part of a family of 11 NIMA kinases involved in cell cycle regulation [9, 10]; while other NEK proteins regulate the formation of mitotic spindle, NEK1, along with NEK10 and NEK11, is known to be involved in DNA damage response [11]. Of note, NEK1 is the only NEK needed to induce DNA damage response through ATM and Rad3‐related (ATR) kinase activation [12]. NEK1 has also been shown to interact with another ALS‐associated protein, C21orf72, during DNA repair; this interaction has been demonstrated to promote the accumulation of NEK1 and neurite outgrowth abnormalities [13, 14]. In vitro, NEK1 deficiency has been shown to result in dysfunctional DNA repair, DNA damage‐induced cell cycle arrest, and mitochondrial function [15, 16, 17]. These impairments can lead to genomic instability, a common feature of and potential contributor to neurodegeneration [18].

The first *NEK1* risk variants discovered were heterozygous loss‐of‐function (LOF) mutations in one whole‐exome sequencing study [19], confirmed by two subsequent studies [4, 6]. This research also revealed the existence of rarer missense variants as well as the incomplete penetrance of LOF *NEK1* mutations [4, 20]. Subsequent investigations have compiled further lists of missense, frameshift, and start codon mutations [21]. We have found three individuals with a *NEK1* mutation through gene panel sequencing in a longitudinal cohort of Scottish ALS patients [22], two with novel heterozygous 5′ splice site mutations, (i) c.214 + 1G > A and (ii) c.1911 + 1 > TATA, and one with a previously characterised heterozygous missense mutation, (iii) c.782G > A, p.Arg261His [4]. We aimed to further characterise these mutations by modelling their predicted protein structures and interrogating their relationship to NEK1 protein expression and TDP‐43 aggregate burden.

## 2 METHODS

### 2.1 Ethics approval

All *post‐mortem* tissue was collected with ethics approval from East of Scotland Research Ethics Service (16/ES/0084) in line with the Human Tissue (Scotland) Act (2006). The use of *post‐mortem* tissue for studies was reviewed and approved by the Edinburgh Brain Bank ethics committee and the Academic and Clinical Central Office for Research and Development (ACCORD) medical research ethics committee (AMREC). Clinical data were collected as part of the Scottish Motor Neurone Disease Register (SMNDR) and Care Audit Research and Evaluation for Motor Neurone Disease (CARE‐MND) platform, with ethics approval from Scotland A Research Ethics Committee (10/MRE00/78 and 15/SS/0216). All patients consented to the use of their data during life.

### 2.2 Mutant identification and in silico modelling

Gene panel sequencing was performed on a longitudinal cohort of Scottish ALS patients, methods for which have been described previously [22]. *NEK1* mutations were analysed as described previously [2] for pathogenicity using maximum entropy modelling (MaxEntScan) and variant effect predictor (VEP) analysis for splice‐site variants, and PolyPhen and Combined Annotation‐Dependent Depletion (CADD) analysis for missense variants. The *NEK1* transcript sequence (ENST00000507142) was referenced using Ensembl [22] and the crystal structure of NEK1 [12] using AlphaFold [23, 24] to annotate splice‐site and missense mutations, respectively.

### 2.3 Immunohistochemistry and BaseScope in situ hybridisation

Brain tissue was taken from standardised Brodmann area (BA) BA4 (i.e., motor cortex) and fixed in 10% formalin for a minimum of 72 h. Tissue was dehydrated in an ascending alcohol series (70–100%), followed by three 4 h xylene washes. Three successive 5 h paraffin wax‐embedding stages were performed followed by cooling and sectioning of formalin‐fixed, paraffin‐embedded tissue on a Leica microtome into 4 μm thick serial sections, collected on Superfrost slides. Sections were dried overnight at 40°C and dewaxed using successive xylene washes, followed by alcohol hydration and treatment with picric acid to minimise formalin pigment and quench lipofuscin. Antigen retrieval was performed in citric acid buffer (pH 6) in a pressure cooker for 30 min, with immunostaining using the Novolink Polymer detection system and anti‐phospho(409–410)‐TDP‐43 antibody at a 1:4000 dilution (CAC‐TIP‐PTD‐MO1) or anti‐NEK1 antibody at a 1:100 dilution (ab186519). Counterstaining was performed using 3,3′‐diaminobenzidine (DAB) chromogen and counterstained with haematoxylin, according to standard operating procedures. BaseScope™ was performed as detailed previously with no additional modifications required [25]. A BaseScope™ probe for *NEK1* was designed and is commercially available from ACDbio.

Digital burden scoring was performed for immunohistochemistry staining using freely available QuPath software implementing superpixel analysis using the following code as described previously [26], with images of steps outlined in Supplementary Figure S1:

setImageType(‘BRIGHTFIELD_H_DAB’); setColorDeconvolutionStains(‘{‘Name’: ‘H‐DAB default’, ‘Stain 1’: ‘Hematoxylin’, ‘Values 1’: ‘0.65111 0.70119 0.29049’, ‘Stain 2’: ‘DAB’, ‘Values 2’: ‘0.26917 0.56824 0.77759’, ‘Background’: ‘255 255 255’}’); setPixelSizeMicrons(0.625,0.625); createSelectAllObject(true); selectAnnotations (); runPlugin(‘qupath.imagej.superpixels.DoGSuperpixelsPlugin’, ‘{‘downsampleFactor’: 1.0, ‘sigmaMicrons’: 3, ‘minThreshold’: 10.0, ‘maxThreshold’: 230.0, ‘noiseThreshold’: 0.0}’); selectDetections(); runPlugin(‘qupath.lib.algorithms.IntensityFeaturesPlugin’, ‘{ ‘downsample’: 1.0, ‘region’: ‘ROI’, ‘tileSizePixels’: 200.0, ‘colorOD’: false, ‘colorStain1’: false, ‘colorStain2’: true, ‘colorStain3’: false, ‘colorRed’: false, ‘colorGreen’: false, ‘colorBlue’: false, ‘colorHue’: false, ‘colorSaturation’: false, ‘colorBrightness’: false, ‘doMean’: true, ‘doStdDev’: false, ‘doMinMax’: false, ‘doMedian’: false, ‘doHaralick’: false, ‘haralickDistance’: 1, ‘haralickBins’: 32}’); setDetectionIntensityClassifications (‘ROI: 2.00 μm per pixel: DAB: Mean’, 0.2689, 0.3007, 1). mRNA count data were generated by counting the number of mRNA binding events (red dots) per cell with 10 cells examined per 3 regions of interest.

### 2.4 Dual chromogen TDP‐43^APT^ and NEK1 immunohistochemical co‐stain

Dual chromogen co‐stain was performed by initially staining the slide as described above for NEK1. Following incubation with DAB chromogen to detect NEK1, the TDP‐43^APT^ staining protocol was implemented in full (https://doi.org/10.17504/protocols.io.eq2lyjo4mlx9/v1) as published previously [27]. The anti‐biotin antibody (used to detect the biotinylated, bound aptamer) was replaced with an alkaline phosphatase‐conjugated antibody (ab6652; diluted 1 in 200 in milliQ water) and FastRed was used instead of DAB chromogen. Slides were counterstained and mounted as described above. This dual staining protocol has been made available online (dx.doi.org/10.17504/protocols.io.14egn6wnml5d/v1) as a detailed SOP.

## 3 RESULTS

### 3.1 Clinical observations reveal varied disease presentation

Clinical data from the Scottish Motor Neuron Disease Register (SMNDR) are summarised in Table 1. Patients 2 and 3 met the El Escorial criteria for Definite ALS and Patient 1 for Probable ALS [28]. Patient 1 noticed left hand weakness, followed by right‐sided limb weakness, and marked weight loss at age 66, and later developed bulbar and respiratory complications, declined intervention, and had a disease duration of 17 months. Patient 1 did not exhibit cognitive or behavioural impairment as measured by the Edinburgh Cognitive and Behavioural ALS screen (ECAS) [3], though had a personal history of anxiety and alcohol dependence. Patient 2 experienced bulbar onset as evidenced by dysarthria at age 57, with an elective gastrostomy insertion at 7 months and a disease duration of 27 months. Patient 2 experienced apathy and loss of empathy, amounting to ALS with behavioural impairment (ALSbi), as determined through ECAS. Patient 2 also had a personal history of anxiety with depression. Patient 3 initially noted progressive weakness of the upper arms at age 70, followed in the same year by facial (but not bulbar) weakness. This was followed over the next 2 years by progressive bulbar symptoms and had a disease duration of 59 months. None of the patients had a family history of ALS, although Patient 1 and 2 had a family history of Alzheimer's disease and schizophrenia, respectively. Upon *post‐mortem* examination, all patients were observed to have vasculopathy. This was accompanied by amyloid angiopathy in Patient 1, and in Patients 2 and 3 vasculopathy was unrelated to amyloid aggregation, though non‐vascular amyloid aggregates and tau tangles were observed.

**TABLE 1:** Summary of clinical phenotypes.

	Patient 1	Patient 2	Patient 3
Risk variant	c.214 + 1G > A (splice)	c.782G > A (missense)	c.1911 + 1 > TATA (splice)
Predicted pathogenicity	LOF	Likely pathogenic	Uncertain significance
Ethnicity	White Scottish	White Scottish	White Scottish
Age of onset (years)	66	57	70
Disease duration (months)	17	27	59
Rate of Disease Progression (decline in ALS‐Functional Rating Score (ALS‐FRS) per month) (*48*—*ALSFRS Score*)*/Months from Onset*	2.9	1.0	No data
Site of onset	Spinal—Upper & lower limb weakness	Bulbar—Dysarthria	Upper limb
Cognitive/Behavioural Impairment	No symptoms of cognitive or behavioural deficit	Yes—ALS with behavioural impairment (ALSbi)	No cognitive deficit (behavioural assessment not done)
El Escorial Classification	ALS Probable	ALS Definite	ALS Definite
Family history (first degree relatives)	Alzheimer's disease	Schizophrenia	Nil of note

### 3.2 In silico modelling suggests distinct mechanisms underpinning pathogenicity of NEK1 variants examined

**FIGURE 1:**
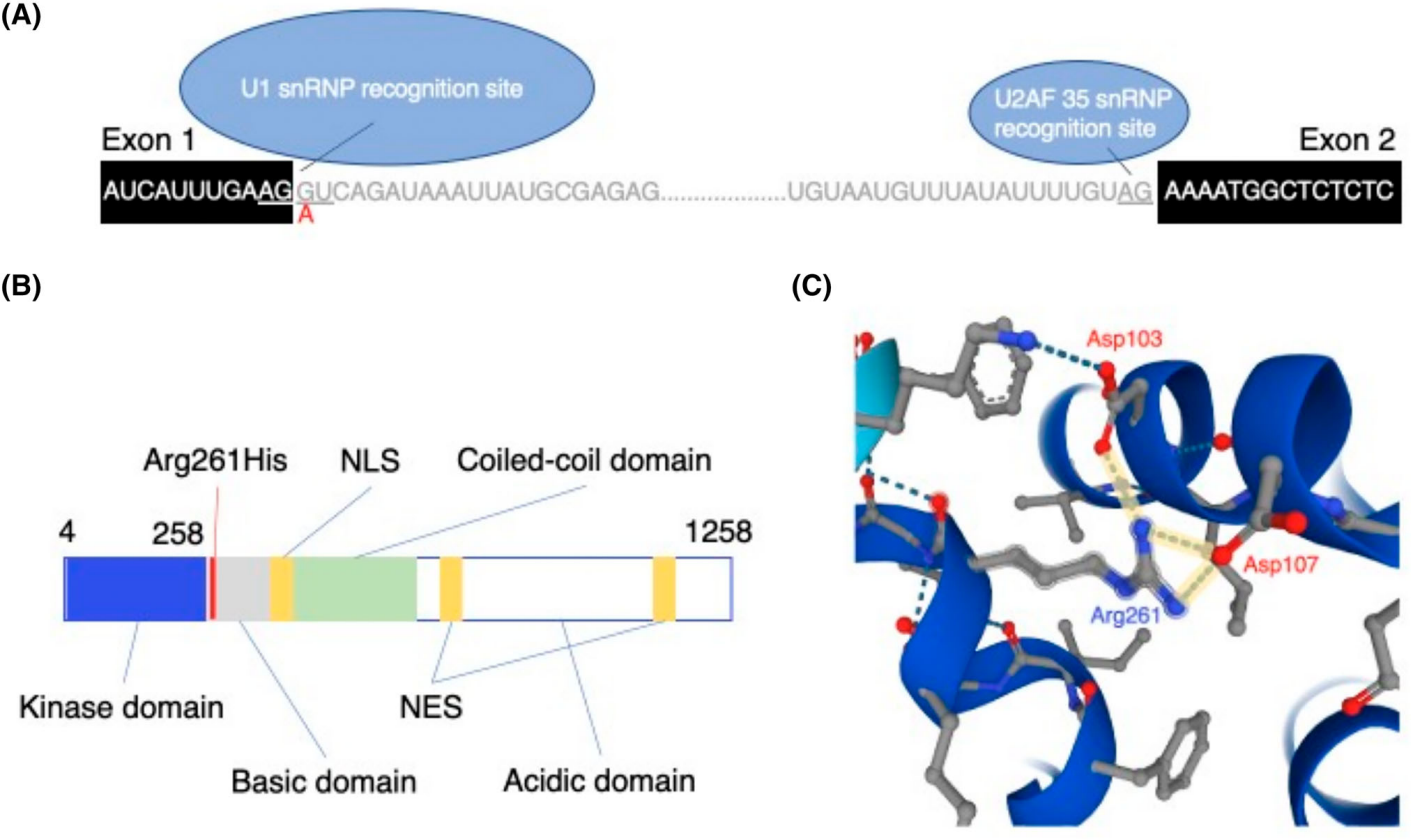
Visualisation of NEK1 mutations in the NEK1 transcript sequence and NEK1 protein structure. (A) Sequences flanking the junction between protein‐coding exon 1 and exon 2 of *NEK1*. The c.214 + 1G > A mutation is indicated in red, and snRNP recognition sites are underlined. (B) Domains of the NEK1 protein with the location of p.Arg261His shown in red. NLS, nuclear localisation signal; NES, nuclear export signal. (C) Crystal structure of NEK1 visualised in AlphaFold. Predicted hydrogen bonding of Arg261 to neighbouring Asp103 and Asp107 is shown in yellow. (D) Sequences flanking the junction between protein‐coding exon 15 and exon 16 of *NEK1*. The c.1911 + 1 > TATA insertion mutation is indicated in red, and snRNP recognition sites are underlined.

The human *NEK1* consensus cDNA sequence is composed of 36 protein‐coding exons, and the human NEK1 protein is formed of a protein kinase domain, a coiled‐coil domain, a basic and acidic domain, one nuclear localisation and two nuclear export signals [29] (Figure 1). The novel 5′, or donor, splice site mutation identified in Patient 1, c.214 + 1G > A, entails a guanine to adenine change one position downstream of the end of protein‐coding exon 4 (Figure 1A). Donor splice site mutations have been known to cause a variety of effects, such as exon skipping, cryptic exon inclusion and intron retention; +1G > A most commonly results in exon skipping. The +1 position is part of a conserved sequence, GU, recognised by the small nuclear ribonucleoprotein (snRNP) 1, after which snRNP1 forms a complex with other snRNPs, including snRNP U2AF 35, which binds to the sequence 5′ of the following exon (i.e., 3′ splice site). While the change from G > A creates a non‐canonical 5′ splice site that could be recognised by snRNP U11, as part of the minor spliceosome, though this would need to be paired by 3′ splice site sequence AC rather than AG [17]. Thus, this mutation could have a negative effect on the recruitment of spliceosome factors. Additionally, as this mutation affects an exon of the protein domain, it could result in an alteration of kinase function. Indeed, c.214 + 1G > A was predicted to be pathogenic (i.e., LOF), with a maximum entropy modelling (MaxEntScan) score difference of 8.18 (reference = 6.80, mutation = −1.38) and variant effect predictor (VEP impact, score = High) analyses of splice‐site mutations [30, 31].

Examination of the crystal structure of NEK1 [12] using AlphaFold [23, 24], demonstrates the predicted hydrogen bonding of Arg261 amino groups with oxygen groups in Asp103 and Asp107 on the neighbouring alpha helix (Figure 1C). In the case of Patient 2, a c.782G > A, p.Arg261His mutation results in a change from an amino acid which is always protonated, to one which titrates at physiological pH, possibly affecting hydrogen bonding. Of note, Asp103 and Asp107 reside in the protein kinase domain, and it is possible that structural alteration in this region may result in functional changes. Indeed, this type of mutation has been shown to alter function in other proteins previously [32]. Furthermore, p.Arg261His was previously predicted to be likely pathogenic, with a Polymorphism Phenotyping (PolyPhen) score of 1 and a Combined Annotation‐Dependent Depletion (CADD) score of 33 [33, 34].

As was the case for Patient 1, Patient 3 also possessed a donor splice‐site mutation. This mutation, 1911 + 1 > TATA, entails the insertion of the sequence TATA before the canonical 5′ splice site motif GU at the +1 position after exon 22 (Figure 1D). Clinically, 1911 + 1‐ > TATA was previously classified as a variant of uncertain significance [2]. While a TATA insertion at the +1 position could interfere with splice site recognition of the spliceosome, it is possible that splicing could still occur after the inserted nucleotides, though this could result in a frameshift leading to abnormal protein product. Indeed, VEP (impact score = High) predicted a frameshift A638fs resulting from this mutation. Maximum entropy modelling also predicted deleterious effects on the splice‐site, with a MaxEntScan score difference of 16.13 (reference = 7.98, mutation = −8.15).

### 3.3 Neuropathological examination reveals distinct putative mechanisms of NEK1 LOF

At *post‐mortem*, cause of death for Patient 1 was noted as respiratory failure secondary to motor neuron disease. Neuropathological examination identified ALS‐related TDP‐43 pathology as well as Braak neurofibrillary tau tangles (stage II) [35], sporadic cerebral amyloid angiopathy, lacunar infarcts, and small vessel disease. Cause of death for Patient 2 was noted as motor neuron disease. Neuropathological examination revealed ALS‐related TDP‐43 pathology, Braak neurofibrillary tau tangles (stage II), amyloid‐beta deposition (Thal phase I) [36], and severe, non‐amyloid small vessel disease. Cause of death for Patient 3 was noted as motor neuron disease. Neuropathological examination identified ALS‐related TDP‐43 pathology as well as Braak neurofibrillary tau tangles (stage II), and small vessel disease.

In‐depth pathological analysis of TDP‐43 aggregate burden was performed across brain regions associated with motor, cognitive and behavioural functions (Table 2). Patients 1 and 3 exhibited only mild neuronal TDP‐43 burden restricted to the primary motor cortex and amygdala. Contrastingly, Patient 2 presented with both neuronal and glial TDP‐43 aggregates in all examined regions, with the highest burden in the motor cortex, the hippocampus, and the amygdala. TDP‐43 aggregates were present in the regions corresponding to the functional impairment each patient experienced, as shown previously in non‐demented ALS cases [19]. However, TDP‐43 aggregates were also present in the amygdala in Patient 1, and areas of executive, language/fluency, sensory, and memory function in Patient 2, despite there being no cognitive impairment in these domains, indicating resilience to TDP‐43 pathology in these areas and specificity but not sensitivity of TDP‐43 pathology to cognitive impairment, as shown previously [19] (see Table 3).

**TABLE 2:** Available TDP‐43 staining data.

Case ID	Cell type	Motor	Executive function					Language				Sensory	Other		
		BA4	BA6	BA11	BA24	BA46	BA9	BA44	BA41	BA20	BA39	BA19	Ant hip	Post hip	Amygdala
Patient 1	Neuronal	1	1	0	0	0	0	0	0	0	0	0	0	0	1
	Glial	0	0	0	0	0	0	0	0	0	0	0	0	0	0
Patient 2	Neuronal	3	3	1	2	2	2	2	2	2	1	2	3	3	3
	Glial	3	2	1	1	2	2	2	1	1	1	1	2	2	2
Patient 3	Neuronal	1	0	0	0	0	0	0	0	0	0	0	0	0	0
	Glial	0	0	0	0	0	0	0	0	0	0	0	0	0	0

*Note*: 1 = mild, 2 = moderate, 3 = severe TDP‐43 pathology [19].

Abbreviation: BA11/12, orbitofrontal cortex; BA19, peristriate cortex; BA20/21, middle and inferior temporal gyri; BA24, ventral anterior cingulate; BA39, angular gyrus; BA4, precentral gyrus (primary motor cortex); BA41/42, transverse temporal area (Heschl's gyri); BA44/45, inferior frontal gyrus (Broca's); BA46, dorsolateral prefrontal cortex; BA6, anterior prefrontal cortex; BA9, medial prefrontal cortex; hip, anterior/posterior hippocampus.

**TABLE 3:** Available cognitive data, tested during life using the Edinburgh Cognitive ALS Screen (ECAS).

Case ID	Language	Fluency	Executive	ALS specific score	Memory	Visuospatial	ALS non‐specific score	ECAS Total	Behaviour
Patient 2	28	20	38	86	18	12	30	116	2 (apathy and empathy)
Patient 3	28	18	42	88	18	12	30	118	Not done

**FIGURE 2:**
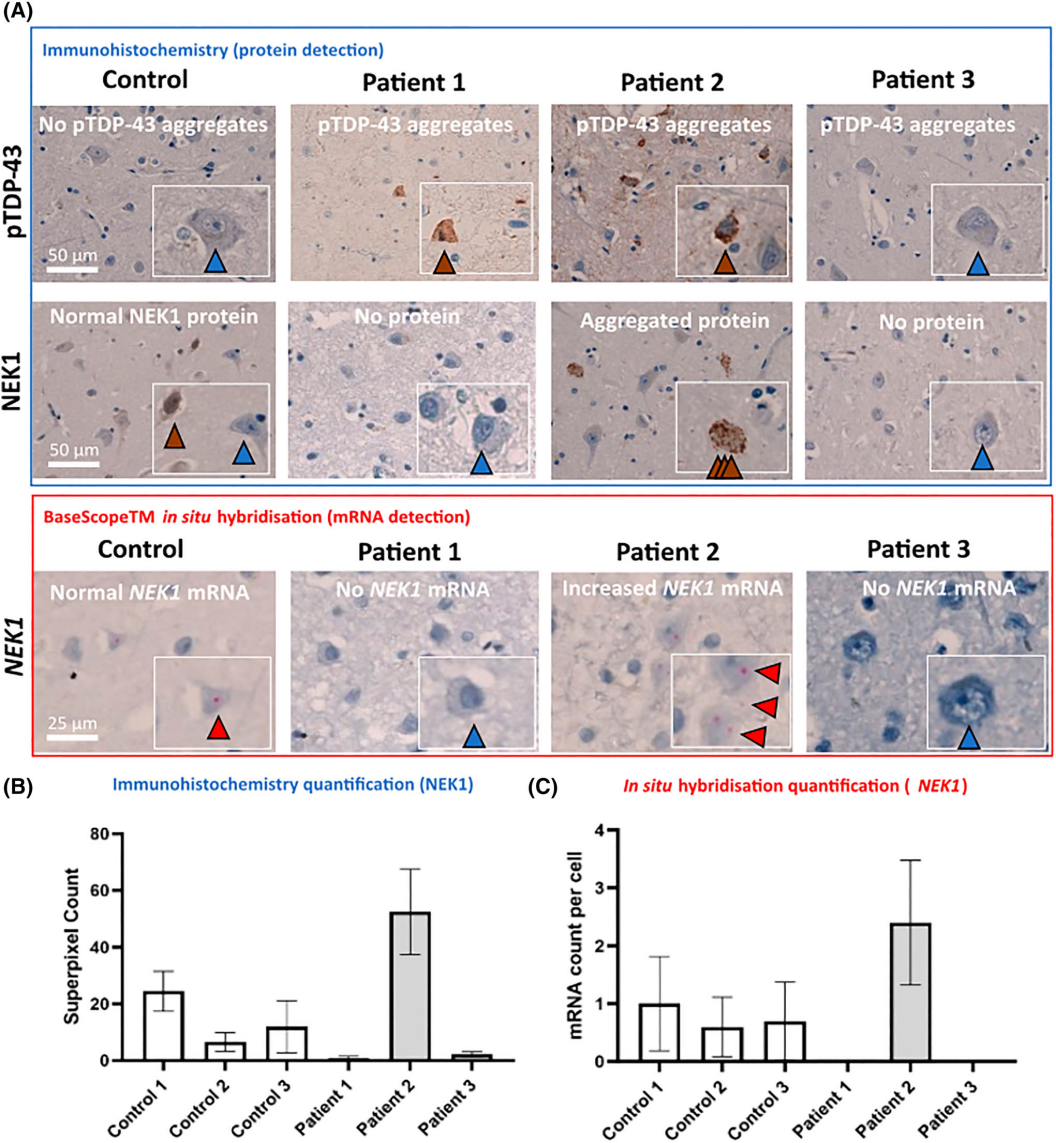
*Histological staining reveals distinct* NEK1 *mutation associated pathology*. (A) ***upper panel*** (***blue outline***) Immunohistochemistry staining of motor cortex demonstrating mild pTDP‐43 aggregation in Patients 1 and 3 but extensive aggregation in Patient 2 (top row); absent NEK1 protein in Patients 1 and 3 and NEK1 aggregates in Patient 2 (second row). Blue arrowhead indicates absence of staining; brown arrowhead indicates increased immunostaining (protein). (A) ***lower panel*** (***red outline***). BaseScope™ in situ hybridisation in motor cortex reveals absent *NEK1* mRNA transcripts in Patients 1 and 3, consistent with LOF through loss of expression, and increased *NEK1* mRNA transcripts in Patient 2 (red box), consistent with compensatory increased expression secondary to NEK1 protein aggregation seen in the upper panel. Red arrowhead indicates increased mRNA transcripts (i.e., each red dot is a single mRNA transcript of NEK1). (B) Quantification of immunohistochemical staining using digital burden scoring employing a superpixel analysis and detection classifier in QuPath. Graphs demonstrate superpixel count based on detection intensity based on a modified Allred score analysis. (C) Quantification of mRNA burden as mRNA counts per cell across 10 cells per randomly generated region of interest.

Tissue from the motor cortex was further analysed with an antibody to NEK1 to assess protein abundance and localisation compared to age‐ and sex‐matched controls. Controls exhibited sparse neuronal‐predominant weak cytoplasmic staining, which was completely absent in Patients 1 and 3 (Figure 2A; quantified in 2b), a finding that corresponded to the absence of *NEK1* mRNA visualised using BaseScope™ in situ hybridisation (Figure 2A; quantified in 2c). Contrastingly, Patient 2 showed extensive neuronal NEK1 protein accumulation in cytoplasmic aggregates, and particularly striking neuropil aggregation mirroring the pattern of TDP‐43 aggregation (Figure 2A). This corresponded to increased numbers of *NEK1* mRNA transcripts (Figure 2A; quantified in 2c). Age‐ and sex‐matched control tissue (*n* = 3) showed minimal NEK1 staining that never occurred as aggregates.

### 3.4 Dual chromogenic staining reveals cells containing both TDP‐43 and NEK1 aggregates

**FIGURE 3:**
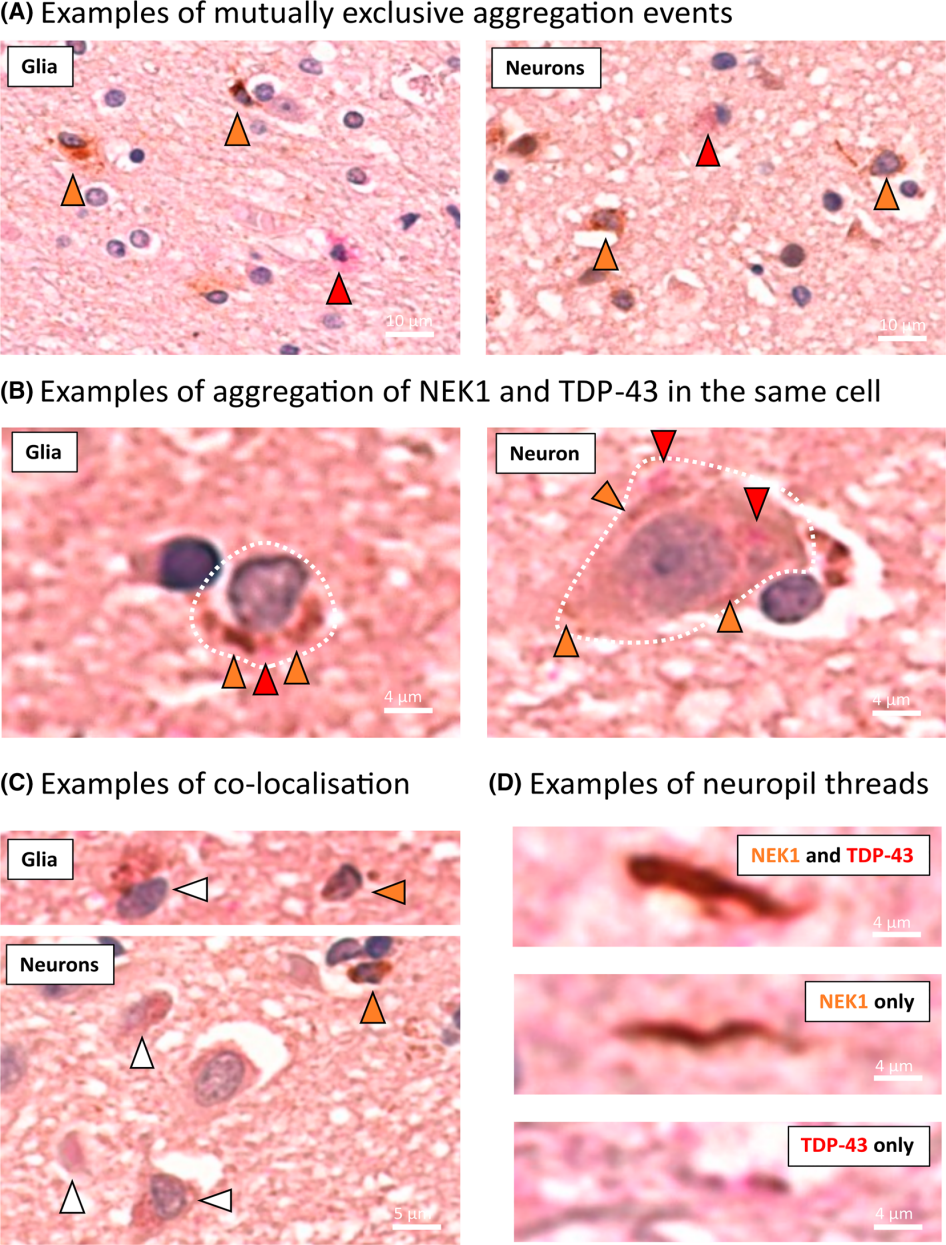
Dual chromogenic staining reveals cells containing both TDP‐43 and NEK1 aggregates. Photomicrographs of motor cortex from Patient 2 stained with TDP‐43^APT^ (red chromogen) and NEK1 (brown chromogen) demonstrating examples of: (A) mutually exclusive aggregation events where red arrowheads indicate cells with only TDP‐43 aggregation and orange arrowheads indicate cells with only NEK1 aggregation. (B) Aggregation of TDP‐43 and NEK1 in the same cells where red arrowheads indicate TDP‐43 positive staining and orange arrowheads indicte NEK1 positive staining. (C) Co‐localisation of TDP‐43 and NEK1, where white arrowheads indicate dual (red and brown) positive staining and orange arrowheads indicate NEK1 (brown)‐only staining for comparison. (D) Staining in neuropil threads, which can be dual positive, solely NEK1 positive, or solely TDP‐43 positve.

As pTDP‐43 and NEK1 antibodies are generated in the same species, it was not possible to perform a dual antibody co‐stain. However, TDP‐43 can also be detected using an RNA aptamer (TDP‐43^APT^) [27], and as TDP‐43^APT^ is generated in vitro there are no concerns regarding species overlap. Using NEK1 IHC and TDP‐43^APT^, we performed a dual chromogen co‐stain, where TDP‐43^APT^ staining can be visualised using red chromogen and NEK1 can be visualised using brown chromogen. For Patient 2, we demonstrate instances of mutually exclusive aggregate staining, where cells exhibit aggregation of either TDP‐43 or NEK1 in both neurons and glia (Figure 3A). There are also instances where TDP‐43 and NEK1 aggregate within the same cell (Figure 3B) and instances where TDP‐43 and NEK1 co‐localise (Figure 3C). We also demonstrate that neuropil threads can be (i) NEK1 and TDP‐43^APT^ dual‐positive, (ii) solely NEK1 positive, or (iii) solely TDP‐43^APT^ positive. Whilst this co‐stain cannot prove that TDP‐43 and NEK1 are co‐aggregating, it does suggest these events co‐occur in the same cell in this patient.

## 4 DISCUSSION

Here we assess a cohort of Scottish patients with differing *NEK1* variants and resulting similarities and differences in clinical and pathological presentations of motor neuron disease. First, all patients exhibited neuropathology unrelated to motor neuron disease, i.e., aggregation of tau and amyloid, in addition to TDP‐43 (in all cases) and NEK1 aggregation (in Patient 2). This may reflect a disruption of proteostasis, perhaps mediated by NEK1 deficiency‐induced impairment of nucleocytoplasmic transport; indeed, sub‐cellular mislocalisation of NEK1 has been shown to lead to genomic instability and affect nuclear pore complex dispersal [37]. All patients also exhibited vasculopathy, accompanied by amyloid angiopathy in Patient 1. This may indicate impairment of blood brain barrier integrity, as seen in a mouse model of *NEK1* deficiency [38], that could be accompanied by inflammation; thus, further investigation into the effects of *NEK1* mutations on inflammatory pathways is warranted.

In terms of *NEK1* transcription and translation, in Patient 1 and 3, *NEK1* RNA and NEK1 protein levels in the motor cortex appeared to be absent compared to controls, indicating that the splice site mutations reported here result in a loss of NEK1 mRNA and protein. In Patient 2, the p.Arg261His mutation may have a similar effect of LOF; however, this could be mediated through NEK1 protein aggregation, rather than a loss of translation. Indeed, Patient 2 exhibited extensive TDP‐43 and NEK1 aggregation in the same cells and accompanied by amyloid aggregates and tau tangles, indicating an aggregation‐prone state. This was also the only patient to exhibit known behavioural impairment, highlighting the need for further investigation into the interplay between NEK1 and TDP‐43 aggregation and how this could contribute to functional impairment in extra‐motor regions. In the future, it will be necessary to further characterise LOF effects of these *NEK1* mutations using functional assays, which cannot be conducted in *post‐mortem* tissue. Additionally, exploration of other markers such as gamma‐H2AX and nuclear pore complex proteins will reveal further detail about the relationship between NEK1 and impaired DNA damage response and nucleocytoplasmic transport, respectively. Finally, analysis of a larger number of patients with *NEK1* variants will be necessary to further characterise the clinical and pathological range of *NEK1*‐related ALS.

In conclusion, despite the small sample size assessed in this case series, we show that TDP‐43 pathology is present in these cases and that NEK1 LOF could be mediated through loss of *NEK1* translation or through aggregation of NEK1 protein as in the case with p.Arg261His mutation, a potential novel pathological feature of NEK1‐ALS.

## AUTHOR CONTRIBUTIONS

All authors contributed to writing the manuscript. All authors contributed to data aquisitiona dn analysis. DJL contributed genetics expertise, JMG contributed pathology expertise.

## FUNDING INFORMATION

This work was supported by NIH (1R01NS127186) to JMG employing JO'S. The Wellcome Trust (108890/Z/15/Z) to OMR. Target ALS (BB‐2022‐C4‐L2) to JMG employing FMW.

## CONFLICT OF INTEREST STATEMENT

The authors declare that there are no conflicts of interest.

## Supporting information


**Supplementary Figure S1.** Superpixel analysis of immunohistochemistry. Representative images along the workflow to achieve digital analysis of immunohistochemical images in Figure 2. These data were obtained using our previously published analysis code [26].

## Data Availability

All data are available within the manuscript. Whole slide scanned images are available and can be obtained upon request from the corresponding author.
