# Supplementary figures and images for: Clinicopathological analysis of NEK1 variants in amyotrophic lateral sclerosis
Source: Brain Pathol. 2024 Jul 10;35(1):e13287. doi: 10.1111/bpa.13287 (PMC11669413; doi:10.1111/bpa.13287)

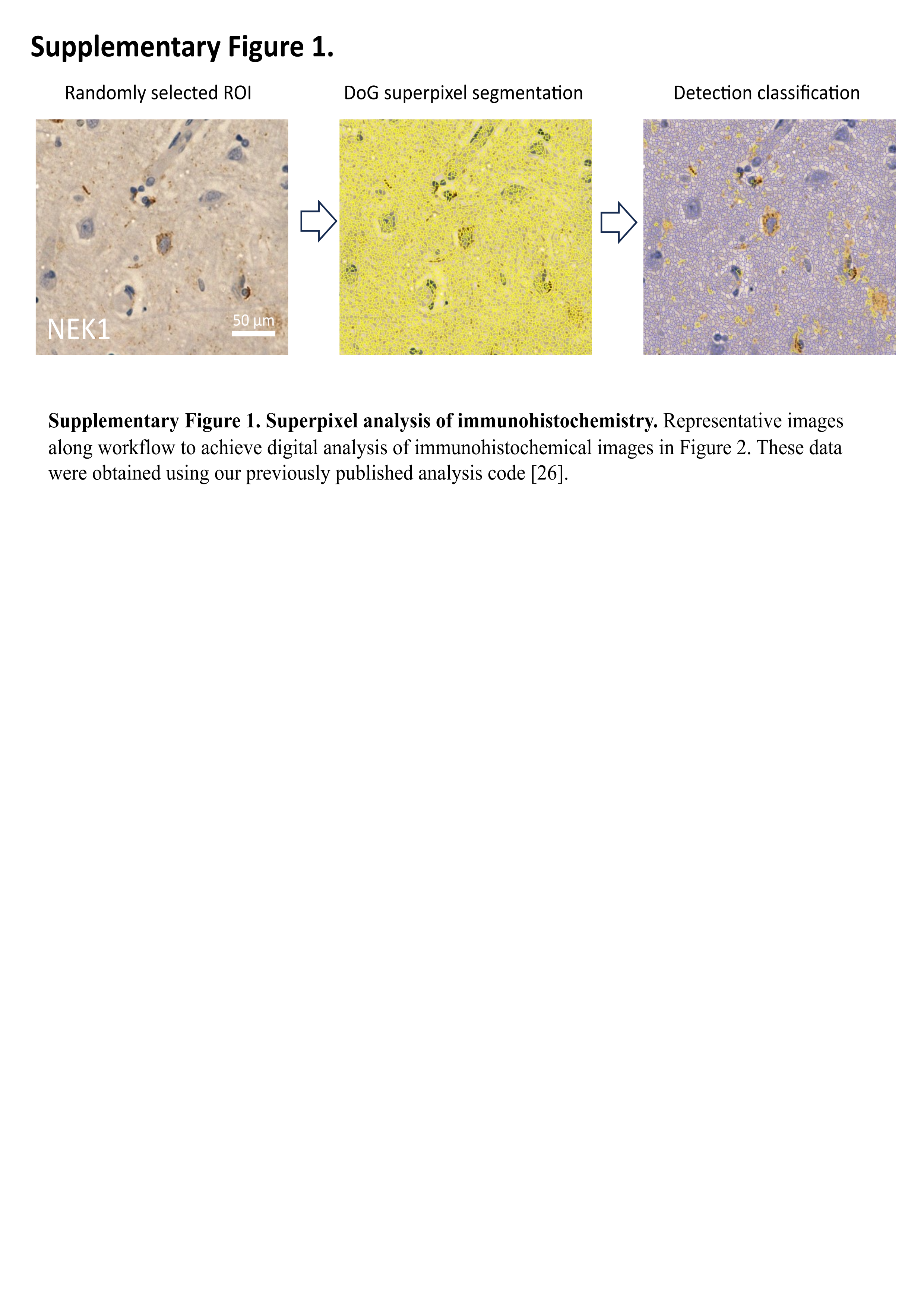

Supplement: Supplementary file 1 — Supplementary Figure S1. Superpixel analysis of immunohistochemistry. Representative images along the workflow to achieve digital analysis of immunohistochemical images in Figure 2. These data were obtained using our previously published analysis code [26]. [file BPA-35-e13287-s001.tiff]
